# The Correlation Between Cervical Proprioception and Scapular Dyskinesis in Patients With Neck Pain: A Case-Control Study

**DOI:** 10.7759/cureus.70869

**Published:** 2024-10-05

**Authors:** Richa R Bisen, Pranaya D Kadam, Annamma Varghese, Rahul Bisen

**Affiliations:** 1 Department of Physiotherapy, K. J. Somaiya College of Physiotherapy, Mumbai, IND; 2 Department of Physiotherapy, Smt. Kashibai Navale College of Physiotherapy, Pune, IND; 3 Department of Musculoskeletal Physiotherapy, K. J. Somaiya College of Physiotherapy, Mumbai, IND; 4 Department of Neurophysiotherapy, Smt. Kashibai Navale College of Physiotherapy, Pune, IND

**Keywords:** cervical proprioception, joint position error test, modified lateral scapular slide test, neck pain, scapular dyskinesis

## Abstract

Introduction

Neck pain is common among office workers, and the assessment of cervical proprioception and scapular dyskinesis is key in the management of patients with neck pain. While some studies have shown the relevance of both factors in neck pain patients, the correlation between the two parameters has not yet been investigated. Hence, this study aimed to determine the correlation between cervical proprioception and scapular dyskinesis in workplace computer users without neck pain (control group) versus those with neck pain (case group).

Methodology

A case-control, correlational study was performed within office settings; based on the selection criteria, 88 participants were included and categorized into two groups consisting of 44 workplace computer users without neck pain in the control group (Group A) and 44 workplace computer users with neck pain in the case group (Group B). Cervical proprioception was evaluated by using the joint position error (JPE) test and scapular dyskinesis was assessed using the modified lateral scapular slide test (MLSST).

Results

There was a statistically significant difference in cervical proprioception and scapular dyskinesis between the case and control groups (p<0.001). Workplace computer users with neck pain showed greater JPE compared to those without neck pain, and scapular dyskinesis was observed in the case group. Moreover, Spearman’s correlation coefficient showed a significant correlation between cervical proprioception and scapular dyskinesis in workplace computer users with neck pain.

Conclusions

The present study provides guidance on the assessment as well as management of JPE with different positions of scapular dyskinesia. The evaluation of scapular dyskinesis is frequent clinically; given its positive correlation, managing JPE in neck pain patients is feasible.

## Introduction

Neck pain is a common musculoskeletal disorder and presents a significant challenge in contemporary everyday life. Neck pain due to prolonged computer use is a frequent issue among office workers, highlighting occupational challenges [1,2]. Proprioception refers to the intricate coordination between sensory and motor signals that govern the body's spatial awareness and precision of movement. Thus, proprioceptors such as the Golgi tendon organ, joint receptor, and muscle spindle receive the afferent signals to maintain the position of the body in space [3-5]. Cervical proprioception is a position sense of the neck and head in space. Cervical proprioception performs an important role in the maintenance of posture and balance. The cervical muscle spindle encompasses numerous intricate proprioceptive receptors crucial for maintaining posture and balance control. However, neck pain leads to diminished cervical proprioception causing joint position error (JPE), disrupting the proprioceptive signals due to the interference caused by neck pain [6-10].

Scapular dyskinesis is defined as the altered scapular position at rest or during upper limb movement. Given the common muscle attachments between the scapula and neck, any alteration in scapular muscle function can contribute to the onset of neck pain [9-12]. Although there is evidence for the relevance of impairment in cervical proprioception and scapular dyskinesis in neck pain patients, there is scarce literature regarding the correlation between the two components [13,14]. Thus, the goal of the present study is to determine the correlation between cervical proprioception and scapular dyskinesis in workplace computer users with neck pain versus those without neck pain. As it is a case-control study, assessing these parameters in asymptomatic computer workers will help us understand if scapular dyskinesis and impairment in cervical proprioception are present sub-clinically. Additionally, this study would help in the assessment of cervical proprioception in neck pain subjects with scapular dyskinesis.

This study aimed to measure cervical proprioception using the JPE test in a case and a control group [5,15] and assess scapular dyskinesis using the modified lateral scapular slide test (MLSST) [16], to find the correlation between cervical proprioception and scapular dyskinesis in both the groups.

## Materials and methods

A case-control, correlational study was performed spanning one year within office settings in Pune, India, after obtaining approval from the Institutional Ethical Committee of Smt. Kashibai Navale College of Physiotherapy (reference no: 12/2023). The inclusion criteria for the case group were as follows: workplace computer users with forward neck posture who work for more than four hours a day [17]; falling in the age group of 18-55 years with neck pain duration of three months or more [10]; pain experienced by the participants in sub-occipital to first thoracic vertebra; and able to perform full shoulder range of motion. The inclusion criteria for the control group were as follows: workplace computer users aged between 18 and 55 years, with no history of neck pain in the past three months. However, cases with traumatic neck injury, cervical prolapsed intervertebral disc, meningitis, cervical spondylosis, and congenital deformities were excluded from the study. Additionally, controls with cervical radiculopathy, cervical spondylolisthesis, congenital deformity, and a history of cervical spine trauma or cervical spine surgery were excluded.

Based on the criteria, a total of 88 participants were enrolled; the case group included 44 symptomatic workplace computer users and the control group included 44 asymptomatic workplace computer users. Participants were chosen using purposive sampling methods to ensure adequate representation from both groups. Demographic information, including sex, age, occupation, duration of computer work, and duration of neck pain (for the case group), was collected from all the participants as part of the study protocol. Cervical proprioception was assessed using the JPE test [5,7,15], while scapular dyskinesis was evaluated using MLSST [12,16]. These assessments were employed to measure specific aspects of cervical and scapular function respectively, providing valuable data for the study analysis.

The JPE test was conducted with the participants seated to minimize the influence of postural compensation on the findings. This seated position helps isolate and more accurately assess cervical proprioception, providing a clearer understanding of the participants' proprioceptive abilities without interference from compensatory movements. The target was placed at a distance of 90 cm on a wall at the height of the subject's head. A lightweight headband with a laser pointer was placed on the head of the participant. The participant was asked to focus on the center of the target. The participant with eyes closed was asked to actively perform movement in one plane of motion and was asked to return to the starting position as accurately as possible and indicate verbally that they had returned to the starting position before opening their eyes. The difference between the starting and end positions was measured and then converted to degrees. Normal cervical proprioception was denoted by error less than 4.5 degrees [5,7,18].

The MLSST can be performed in a standing or sitting position. The test was performed by first palpating the spinous process of the T7 vertebra. Then, the distance between the inferior angle of the scapula and the spinous process was measured in three different arm positions such as P1 arm by the side, P2 hands on hips, P3 90 degrees shoulder abduction in scaption plane with maximum internal rotation and 1 kg weight in hands. All the measurements were done bilaterally. A difference of more than 1.5c m suggested positive results [12,16].

Statistical analysis

Normality was checked by using the Shapiro-Wilk test, which suggested that the data was non-normal. Thus, for comparison between groups, the Mann-Whitney U test was utilized and Spearman’s correlation coefficient was estimated. SPSS Statistics version 26.0 (IBM Corp., Armonk, NY) and Minitab software were used for statistical analysis.

## Results

The mean age of the participants in the control group was 33.66 ±8.52 (19-54) years while that in the case group was 33.68 ±8.23 (23-55) years, with a median value of 32.50 years and 30.50 years respectively. Additionally, in the control group, the majority of participants were female (n=23, 52.27%) while males accounted for 47.72% (n=21). Similarly, in the case group, most of the participants were female (n=26, 59.09%) while males constituted 40.90% (n=18). Moreover, the mean duration of computer work in the control group was 8.114 ±0.970 (7-10) hours with a median value of 8.000 hours; in the case group, it was 8.273 ±1.042 (6-10) hours with a median value of eight hours. Furthermore, the mean duration of neck pain among the participants in the case group was 5.636 ±2.633 (3-12) hours with a median value of 5.000 hours.

A significant difference was observed between the case and control group in the JPE of left lateral rotation, flexion, right lateral rotation, and extension (p<0.001). Additionally, the results suggested that JPE was greater in workplace computer users with neck pain. Moreover, there was a statistically significant difference in scapular dyskinesis between the two groups (p<0.001), which suggested the presence of scapular dyskinesis in the case group, as depicted in Table 1.

**Table 1:** Comparison of parameters between the groups *Statistically significant (two-tailed) SD: standard deviation

Variable	Groups	Mean ±SD	P-value
Flexion	Group A	2.932 ±0.966	<0.001*
	Group B	5.9 ±1.61	
Extension	Group A	2.907 ±0.932	<0.001*
	Group B	6.525 ±1.7038	
Right lateral rotation	Group A	3.098 ±0.876	<0.001*
	Group B	7.618 ±1.583	
Left lateral rotation	Group A	3.409 ±0.702	<0.001*
	Group B	7.395 ±1.55	
Scapular dyskinesis P1	Group A	0.0455 ±0.2107	<0.001*
	Group B	1.3636 ±0.3984	
Scapular dyskinesis P2	Group A	0.2318 ±0.4334	<0.001*
	Group B	1.7295 ±0.2119	
Scapular dyskinesis P3	Group A	0.3886 ±0.5253	<0.001*
	Group B	1.8136 ±0.2417	

**Figure 1:**
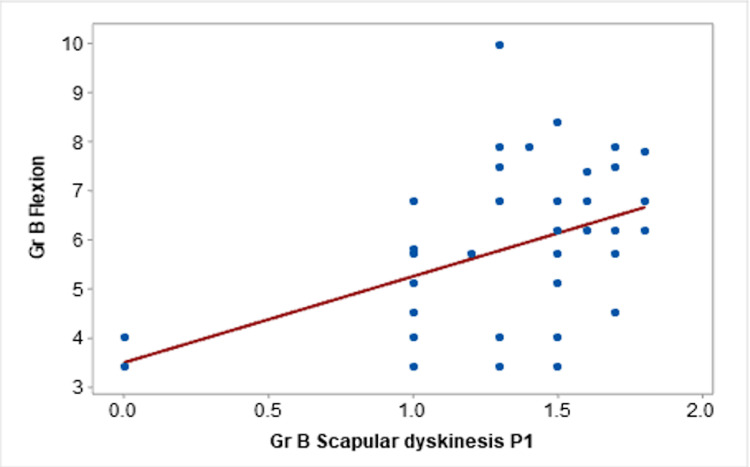
Correlation of scapular dyskinesis P1 with flexion in Group B

Moreover, Spearman’s correlation coefficient for case group (Group B) suggested that there was a moderate positive correlation between the P1 position of MLSST and the flexion component of the JPE test (r=0.404) as illustrated in Figure 1.

**Figure 2:**
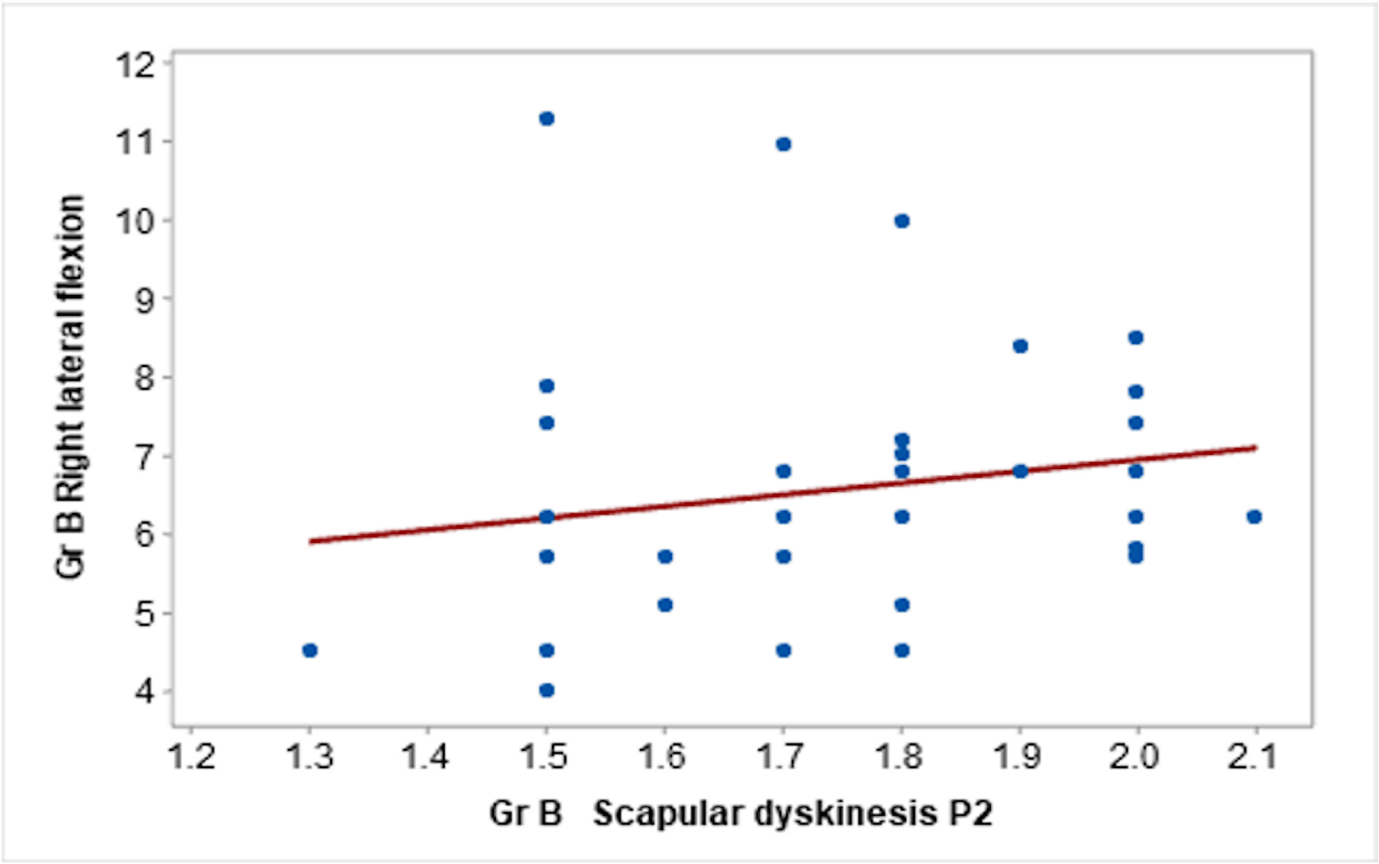
Correlation between scapular dyskinesis P2 and right lateral flexion in Group B

Additionally, there was a moderate positive correlation between the P1 position of MLSST and the extension component of the JPE test (r=0.426). The P2 position of the MLSST demonstrated a moderate correlation with both left (r=0.597) and right (r=0.365) lateral flexion, as shown in Figure 2.

**Figure 3:**
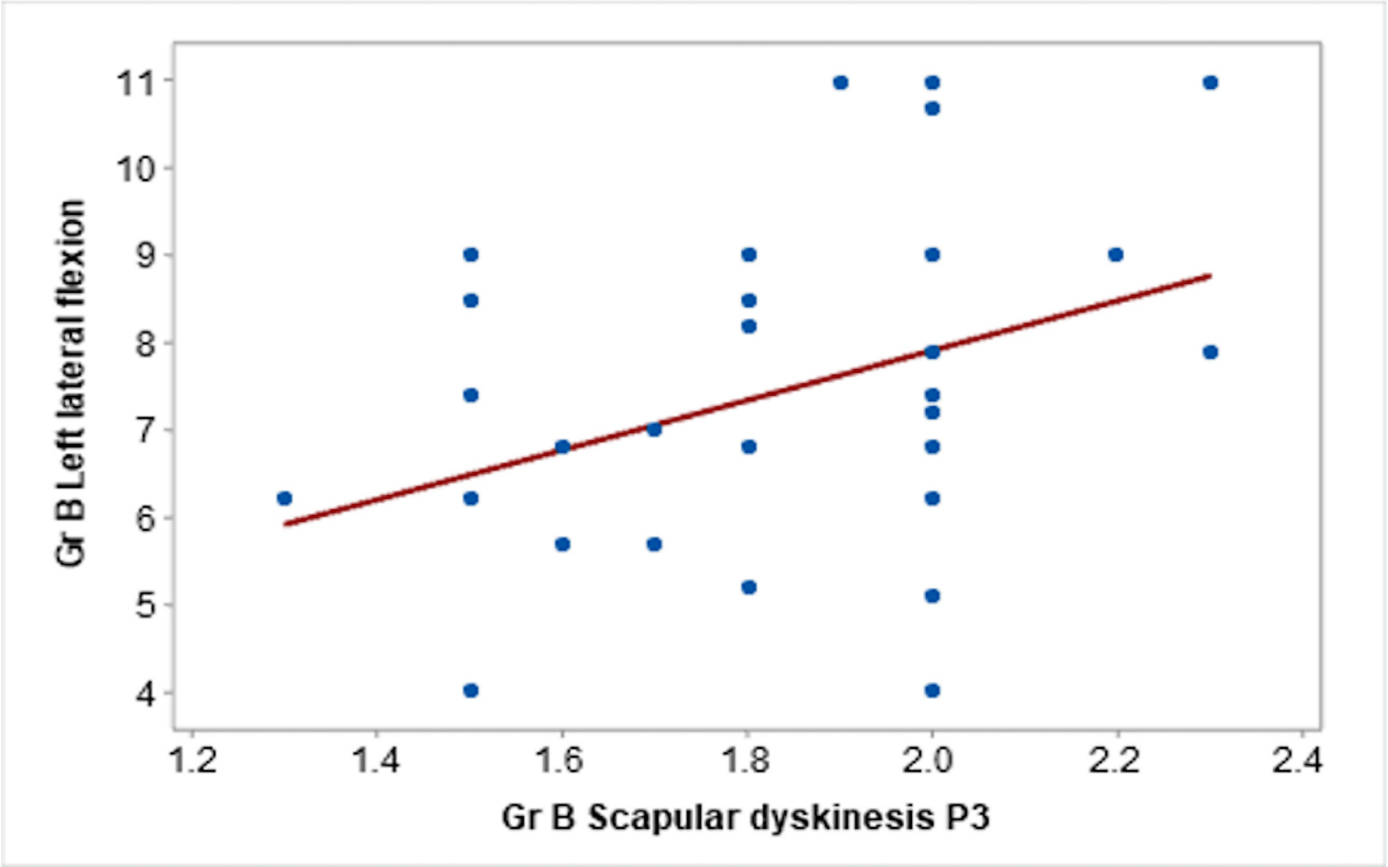
Correlation of scapular dyskinesis P3 with left lateral flexion in Group B

The P3 position exhibited a moderate correlation between the P3 position and right lateral flexion (r=0.400), and a strong correlation with left lateral flexion (r=0.614), as depicted in Figure 3.

Furthermore, for the control group, the results suggested that there was a weak, insignificant positive correlation between all components of JPE with three positions of scapular dyskinesis, as shown in Table 2.

**Table 2:** Correlation between all components of JPE with three positions of scapular dyskinesis in the control group JPE: joint position error

Group A (n=44)		Scapular dyskinesis P1	Scapular dyskinesis P2	Scapular dyskinesis P3
Flexion	r-value	0.252	0.021	-0.031
	p-value	0.099	0.895	0.842
Extension	r-value	0.169	0.102	0.108
	p-value	0.272	0.512	0.484
Right lateral flexion	r-value	0.000	0.146	0.262
	p-value	1.000	0.343	0.086
Left lateral flexion	r-value	-0.123	0.005	-0.075
	p-value	0.427	0.974	0.630
Right lateral rotation	r-value	0.237	0.115	0.096
	p-value	0.122	0.456	0.535
Left lateral rotation	r-value	0.205	0.174	0.068
	p-value	0.181	0.259	0.662

## Discussion

The current study assessed the outcomes of cervical proprioception and scapular dyskinesis within two groups and engaged in an exploration of the correlation between these measured variables. Cervical proprioception was measured using the JPE test in both groups and the results suggested that there was a statistically significant difference in the cervical proprioception of both groups (p<0.001). Cervical JPE was greater in workplace computer users with neck pain in comparison to workers without neck pain. These findings align with those of previous similar studies involving neck pain patients [6]. A significant difference was observed in all three positions of MLSST between the two groups (p<0.001) and scapular dyskinesis was observed in the case group during the study.

We found a moderately significant positive correlation between the P1 position of MLSST and JPE of flexion as well as extension degrees, which could be attributed to the fact that workplace computer users hold sitting posture for prolonged periods, which is associated with poor ergonomics, inadequate lighting condition, inappropriate height of chair and desk, and improper arrangement of visual display unit leading to altered postural behavior [1,19]. Hence, these workers tend to attain increased thoracic kyphosis or slouched posture, resulting in inappropriate activation of stabilizers of scapula such as serratus anterior, lower trapezius, rhomboids, middle trapezius, with neck in flexion or extension for prolonged periods, which decreases the efficiency of muscles inputs [20-24]. This altered muscle activity puts a detrimental load on the cervical spine. Additionally, neck pain interferes with the afferent signals from the proprioceptors, causing inappropriate proprioceptive information. Neck pain reduces the specificity of muscle activation, and causes dysfunction in coordination [6]. Thus, inappropriate biomechanics and altered proprioceptive inputs might be the reason for the correlation between the two.

A significant positive correlation between the P2 position of MLSST and JPE of left rotation as well as right rotation was observed. Likewise, there was a significant positive correlation between the P3 position of MLSST with JPE of right and left rotations. This could be due to the movement impairment associated with shoulder abduction. When there are alterations in muscle activity during shoulder abduction, the scapula tends to rotate downwardly if there is insufficient upward rotation activity in the levator scapulae and trapezius muscles. It occurs due to the force imbalance between the upper as well as lower trapezius and increased stiffness of levator scapulae and rhomboids which impedes the upward rotation scapula [20,25]. Thus the muscle inactivity of the upper trapezius and stiffness in levator scapulae which assists in neck rotation, along with flexibility deficit in surrounding muscles as well as pain signals might hinder the proprioceptive inputs, thereby causing JPE [26].

Our analysis of the correlation in the control group suggested that there was no correlation between JPE and scapular dyskinesis as there was no JPE or scapular dyskinesis in the control group. These findings address our query as to whether both these parameters are present sub-clinically.

Strengths and limitations

A positive correlation between scapular dyskinesis and JPE in neck pain participants was observed, which suggests that if scapular dyskinesis is seen in the P1 position of modified scapular slide test, assessment and management of JPE of cervical flexion as well as extension can be done. Similarly, if scapular dyskinesis is seen in P2 and P3 positions, assessment and management of JPE of left and right lateral rotation can be performed. However, the study has certain limitations, which involved outcome measures for posture assessment, utilization of other sensitive instruments like cervical range of motion device for cervical proprioception measurement, and other confounding factors like ergonomics; moreover, workplace conditions were not matched between the two groups.

## Conclusions

Our findings demonstrated a positive correlation between cervical proprioception and scapular dyskinesis in workplace computer users with neck pain and no correlation between cervical proprioception and scapular dyskinesis in those without neck pain. Thus, the present study guides the assessment as well as management of JPE with different positions of scapular dyskinesis. The assessment of scapular dyskinesis is frequent in clinical settings. Given its positive correlation, managing JPE in neck pain patients becomes feasible. We recommend further studies involving gender-wise correlation of proprioception impairments and scapular dyskinesis as well as professionals in various other fields and age groups.
